# Rutting Behavior of Dual-Layer Asphalt Pavements Subjected to Variable Temperature

**DOI:** 10.3390/ma18112603

**Published:** 2025-06-03

**Authors:** Ya Tan, Yingjun Jiang, Chenfan Bai, Hongjiang Zhang, Yingchao Liang, Wenhui Lou, Zhejiang Chen

**Affiliations:** 1 Key Laboratory for Special Area Highway Engineering of the Ministry of Education, Chang’an University, Xi’an 710064, China; 2 Shaanxi Transportation Holding Municipal Road & Bridge Group Co., Ltd., Xi’an 710064, China; 15829932171@163.com (H.Z.); liangyc0523@163.com (Y.L.); 3 Jinhua Communications Investment Group Co., Ltd., Jinhua 321002, China

**Keywords:** asphalt pavement, rutting behavior, loaded-wheel tracking test, variable temperature, actual service condition

## Abstract

Traditional laboratory rutting tests are performed at a constant temperature by neglecting pavement temperature variation. The mechanical properties of asphalt are susceptible to temperature variation. This sensitivity to temperature variations significantly influences the performance and durability of asphalt pavements. Following this purpose, a stepwise temperature-controlled rutting test method was proposed to investigate the rutting development of double-layer asphalt pavement (DLAP) under variable temperature. A time-hardening model was developed and employed to evaluate the rutting performance of DLAP under variable temperature. Results indicate that the rutting development of DLAP exhibits a stepwise variation when subjected to variable temperatures. Within a specific constant temperature range, rutting development can be fitted using a power function of load cycles. The rutting deformation of DLAP predominantly occurs at 20 °C; once the temperature exceeds 50 °C, the rutting development accelerates and becomes difficult to stabilize. The time-hardening model effectively captures the rutting development under variable temperature. The predicted values align closely with field values, which demonstrates the model’s feasibility in calculating rutting deformation under variable temperature. Under actual service conditions, the rutting development of DLAP follows a periodic S-shaped growth, yet this trend can still be represented by a power-law function. DLAP exhibits satisfactory durability and structural stability, effectively addressing the challenges posed by traffic loads and high temperatures in test sections.

## 1. Introduction

Asphalt pavements, which exhibit thermoplastic properties and notable heat absorption capacity, are susceptible to permanent plastic deformation, particularly during periods of high temperature [1,2,3]. Such permanent deformations accumulate progressively under repeated vehicle loads, forming longitudinal grooved patterns on the pavement, known as rutting [4]. Factors contributing to rutting include pavement structure, layer thicknesses, material characteristics, environmental conditions, and the distribution, volume, and composition of wheel loadings [5]. With global warming and increasing traffic volumes, the rutting issue of asphalt pavements is becoming more prominent, which considerably increases the risk of traffic accidents [6,7]. Previous work has suggested that the actual causal mechanisms through which pavements deteriorate may be far more complex, with geometries affecting the way in which people drive, which in turn affects the distribution of loads on the pavement surface, causing deterioration. In the case of the most common form of structural deterioration of flexible asphalt pavements—rutting—it is also suggested that feedback loops might occur between rutting and driver behaviors [8]. In China, nearly 80% of asphalt pavement maintenance can be attributed to rutting; meanwhile, the development, prediction, and prevention of rutting have become important topics in the field of road engineering [9].

Rutting development in asphalt pavements can generally be divided into three stages. In the first stage, the asphalt mixture gradually densifies under loads, with rutting deformation increasing as a power-law function. This initial densification is primarily due to inadequate compaction of the asphalt mixture [10]. During construction, factors such as aggregate size, gradation type, and degree of compaction significantly influence rutting deformation in this stage [11]. In the second stage, the asphalt mixture is in a quasi-static state with constant flow. The growth rate of rutting deformation remains nearly constant, directly reflecting the pavement’s capacity to resist permanent deformation [12]. In the third stage, the internal structure of the asphalt mixture becomes unstable, leading to shear failure [13]. Asphalt pavements are typically repaired before rutting deformation reaches the third stage to prevent damage to the structural layer and ensure traffic safety. Therefore, many empirical rutting models employ a power-law function to describe the development of rutting deformation with the load cycles [14].

Current research provides a relatively comprehensive understanding of rutting development in asphalt pavements. However, there are still two aspects that require further enrichment and supplementation. A notable point is a limited focus on the rutting behavior of asphalt pavement under variable temperature [9]. Most laboratory studies utilize traditional wheel-tracking tests to investigate rutting behavior. During these tests, the temperature is kept constant, neglecting the cyclic variation in pavement temperatures under actual service conditions [15]. This leads to a research focus on constant temperatures, creating a gap in understanding the rutting behavior under actual service conditions. Although few studies have reported the rutting behavior of asphalt pavements under variable temperature, these investigations are confined to numerical simulations [16,17,18]. Therefore, it is essential to conduct experimental research to validate and extend existing findings.

Furthermore, laboratory research on the rutting behavior of multi-layer asphalt pavement is limited. Traditional rutting tests typically employ single-layer rutting specimens to evaluate rutting behavior. However, asphalt pavements consist of multiple layers with different mixture types and thicknesses. Several research studies indicate that structural combinations play an important role in the road performance of asphalt pavements [19,20,21,22]. Single-layer rutting specimens cannot represent actual pavement structures and interlayer interactions, potentially compromising the reliability of assessment results. To address these limitations, a multi-layered rutting specimen incorporating various asphalt mixtures should be recommended [16,23] Such rutting specimens can more closely represent the actual structure of asphalt pavements, providing more reliable evaluations of rutting behavior.

This article concentrates on the rutting development and performance of asphalt pavements under variable temperature. To address this, we conducted laboratory and field tests on the Nande Highway in China. Firstly, dual-layer rutting specimens consistent with field pavement structure were prepared using the laboratory roller-compaction method. Next, a stepwise temperature-controlled rutting test method was proposed to investigate the effect of temperature variation on rutting development. Additionally, a time-hardening model was developed to estimate rutting deformation under variable temperatures, and the reliability of this model was validated through field experiments. Finally, the rutting performance of double-layer asphalt pavement (DLAP) was evaluated based on actual pavement temperatures and traffic conditions. The results can provide a useful reference for the design and maintenance of asphalt pavements.

## 2. Materials and Methods

### 2.1. Materials

#### 2.1.1. Asphalt

The binder in the asphalt mixture was 70# petroleum asphalt produced in Ningbo, China. Styrene–butadiene–styrene (SBS)-modified asphalt, produced in Karamay, China, was employed as the tack coat to provide interlayer bonding. The technical properties of the asphalts are listed in Table 1.

#### 2.1.2. Aggregate

The aggregates for the asphalt mixture were obtained from a quarry in Shangluo, China. Table 2 lists the technical properties of the aggregates, tested following the Chinese specification JTG 3432-2024 [24].

#### 2.1.3. Gradation of the Asphalt Mixture

The surface and bottom layers of the DLAP are composed of AC-16 and AC-20 asphalt mixtures, respectively, where AC-16 and AC-20 refer to asphalt mixtures with a nominal maximum aggregate size of 16 mm and 20 mm, respectively. According to the Chinese specification JTG F40-2004 [25], the mineral aggregate gradations are designed as shown in Table 3.

The optimum asphalt content (OAC) of the two asphalt mixtures was determined using the Marshall design method, with the results presented in Table 4. This method determines the OAC through an assessment of the impact of asphalt content on the mechanical properties and volumetric parameters of the two asphalt mixtures.

### 2.2. Specimen Preparation Method

**Figure 1:**
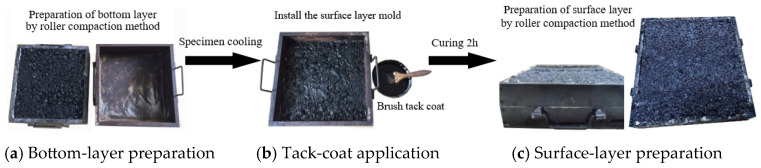
Preparation of dual-layer rutting specimens.

Dual-layer rutting specimens are rectangular with side lengths of 30 cm and a thickness of 10 cm. They consist of a 3 cm thick surface layer and a 7 cm thick bottom layer. The specimens were prepared through laboratory roller compaction, referring to the Chinese standard JTG E20-2011 [26]. The preparation of the specimens involved a three-step process, as depicted in Figure 1.

(1) Bottom-layer preparation: According to the mold volume and maximum dry density of the asphalt mixture, the mass of the asphalt mixture in the loose state was determined. Then, the mixture was poured into the bottom-layer mold and compacted to the mold height using a roller compactor. The formula used to determine the mass of the asphalt mixture in the loose state is presented as Equation (1):
  (1) $$m_{l}=\rho_{d,\max}V$$
  where *m_l_* is the mass of the asphalt mixture in the loose state, *ρ*_d,max_ is the maximum dry density of the asphalt mixture, and *V* is the mold volume.
(2) Tack-coat application: After the bottom layer cooled, the tack coat was uniformly applied to it at an application rate of 0.45 kg/m^2^. In order to fully penetrate and cure the tack coat, the surface-layer mold was positioned after a two-hour curing period for the tack coat.
(3) Surface-layer preparation: The mass of the asphalt mixture in the loose state was determined using Equation (1). Then, the asphalt mixture was poured into the surface-layer mold and compacted to the mold height using a roller compactor.

### 2.3. Rutting Test Method

According to the Chinese specification JTG E20-2011 and JTG D50-2017 [26,27], the loaded-wheel tracking test was utilized to investigate the rutting behavior of double-layer asphalt pavements. Under realistic conditions, pavement temperatures exhibit three variable states: constant, increasing, and decreasing. Accordingly, rutting tests were conducted under the following conditions: (i) Constant temperature, wherein a specific temperature was maintained throughout the test; (ii) upward stepwise temperature, wherein the temperature incrementally increased from 20 °C to 70 °C in steps of 10 °C, and a 3 h load was applied at each temperature level; and (iii) downward stepwise temperature, wherein the temperature incrementally decreased from 70 °C to 20 °C in steps of 10 °C, and a 3 h load was applied at each temperature level.

Before rutting testing, each dual-layer rutting specimen was preheated for 8 h to ensure a uniform internal temperature and to reach the required test temperature [28]. A rubber wheel, with a diameter of 200 mm and a width of 50 mm, rolled over the specimen at a frequency of 42 times/min, exerting a 0.7 MPa load to simulate vehicle movement. The rutting deformation of the specimens was recorded using sensors installed on the rubber wheels, with the load maintained for 3 h at each temperature level. Subsequently, the test temperature was changed, the loading was paused, and the specimens were preheated for 8 h. Three parallel specimens were used for each test. When the coefficient of variation in the final rutting deformation of specimens did not exceed 20%, the average value was adopted as the test result. Otherwise, additional parallel specimens were added until the coefficient of variation was below 20%.

### 2.4. Field Measurement Method

The field test was conducted to collect the pavement temperature and rutting deformation on the Wuwei section (117°43′ N, 31°11′ E) of the Nande Highway in China. The section spans 1 km and adopts a double-layer asphalt pavement structure, consisting of a 3 cm thick AC-16 surface layer and a 7 cm thick AC-20 base layer.

#### 2.4.1. Pavement Temperature Measurement

**Figure 2:**
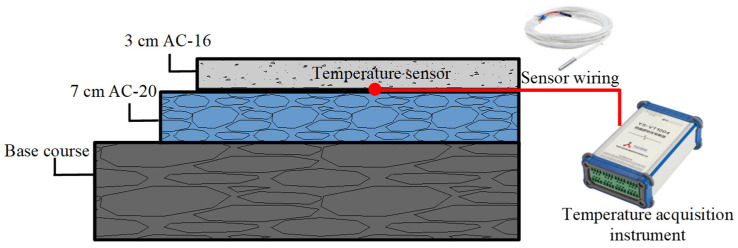
The pavement temperature acquisition system.

The pavement temperature was measured by the temperature sensors and a temperature acquisition device. The temperature sensor utilized a 3 K thermistor treated with waterproof sealing to prevent interference from external environmental conditions. The temperature acquisition device collected the electrical signals transmitted by the sensor at hourly intervals, subsequently converting these signals into digital form for recording. The configuration of the pavement temperature acquisition system is depicted in Figure 2.

#### 2.4.2. Rutting Deformation Measurement

Rutting deformation of the asphalt pavement was measured using a vehicle-mounted laser profilometer, which was manufactured in Chengdu, China. Rutting deformation monitoring commenced after the field test section opened to traffic in September 2021. The rutting deformation was continuously measured at 15-day intervals. During each measurement, rutting deformation data were measured every 10 m, and the measured result was taken as the average rutting deformation across the entire section. Due to the long duration of the actual field measurements, using the data collected from the field tests, we have fully considered the effects of rutting deformation in different seasons and temperature cycles.

## 3. Results and Discussion

### 3.1. The Effect of Temperature Variation on Rutting Development

#### 3.1.1. Constant Temperature State

**Figure 3:**
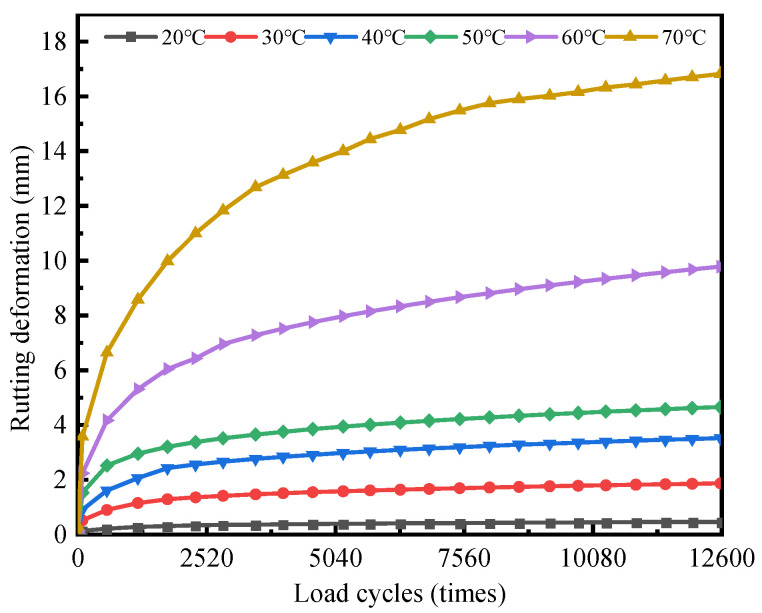
Rutting development curve at different constant temperatures.

Figure 3 shows the rutting development at different constant temperatures.

The rutting deformation gradually increases and stabilizes as load cycles increase, without reaching the third stage of shear damage. Approximately 70% of the rutting deformation was generated during the initial 2520 load cycles. Current research indicates that early rutting is concentrated within the surface layer, mainly caused by compressive deformations in the asphalt mixture [10,22]. Improving the compaction level can effectively mitigate early rutting disease in asphalt pavement. As rutting develops, rutting deformation increasingly occurs in the bottom layer, where shear stresses are more pronounced. The contribution of the bottom layer to total rutting deformation gradually increases, eventually accounting for 52% to 60% of the overall rutting deformation [29].

Asphalt mixtures are temperature-sensitive materials, displaying varying deformation characteristics at different temperatures [20,21,22,23,24,25,26,27,28,29,30,31,32]. DLAP, composed of asphalt mixtures, also retains this property. The test results indicate that the rutting development of DLAP is sensitive to temperatures of 20 °C and 50 °C. When the temperature is below 20 °C, the rutting deformation of DLAP is small. The rutting development stopped at about 2520 loading cycles, with the rutting deformation finally stabilized at 0.4 mm. Between the temperatures of 20 °C and 50 °C, the rutting deformation of DLAP develops slowly, with the deformation rate eventually stabilizing at a range from 1.3 to 3.4 × 10^5^ times/mm, resulting in a final deformation consistently under 5 mm. Rutting deformation increases significantly above 50 °C. As the temperature increases from 50 °C to 60 °C and 70 °C, the final rutting deformation, respectively, reaches up to 9.6 and 16.8 mm, with each increment of 10 °C resulting in a 72.6–97.9% increase in the rutting deformation. These findings are consistent with the conclusions of Pouranian’s study [33].

Rutting development under constant temperature can be briefly described using the power-law model [34], which is shown in Equation (2):

(2) $$RD=\alpha T^{\beta }N^{\gamma }$$

where *RD* is the rutting deformation; *T* is the pavement temperature; *N* is the number of load cycles; and *α*, *β*, and *γ* are material coefficients.

The rutting development curves were fitted to obtain the following parameters: *α* = 1.15 × 10^−6^, *β* = 3.27, and *γ* = 0.28. The regression results show a high goodness of fit (R^2^ = 0.98, *p* < 0.05), suggesting that the power-law model effectively characterizes rutting development under constant temperature, which is consistent with the rutting development regularity, which is shown in Appendix A.

#### 3.1.2. Increasing Temperature State

**Figure 4:**
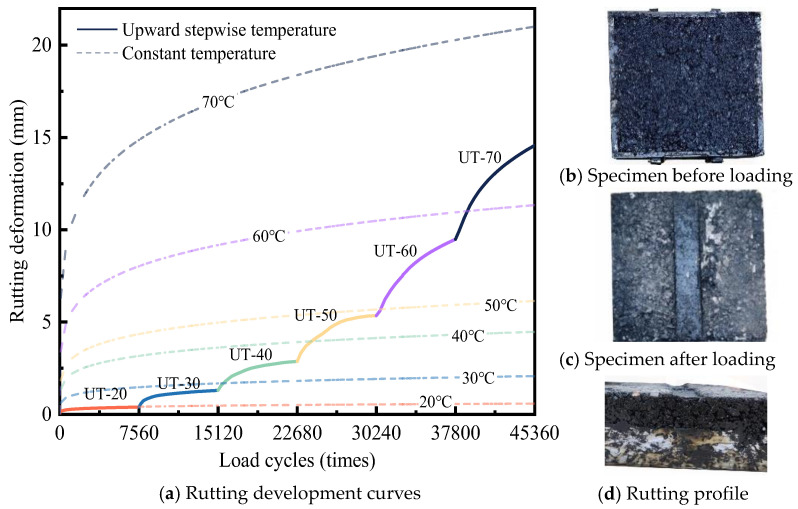
Rutting test results under upward stepwise temperature.

Figure 4 shows the rutting test results under upward stepwise temperature, where UT-70 indicates an increase in temperature up to 70 °C.

Figure 4a shows that each temperature rise considerably escalates the rutting deformation. This trend persists even after rutting deformation has stabilized, indicating that any further increase in temperature inevitably results in additional deformation. When the temperature is <50 °C, the rutting deformation of DLAP gradually stabilizes, with a final deformation rate exceeding 10^5^ times/mm. Exceeding this threshold will result in a substantial reduction in the DLAP rutting resistance, causing a surge in rutting deformations and making it difficult to restabilize as the temperature increases. This observation reiterates the sensitivity of asphalt-pavement rutting to the threshold of 50 °C. Notably, except for the rutting development at UT-20, which closely aligns with the rutting results obtained under constant temperature, the rutting deformation rates at other temperature intervals are generally slower than those at constant conditions. Taking 40 °C as an example, DLAP experiences 7560 load cycles, resulting in a rutting deformation of 1.6 mm at UT-40, compared to 3.2 mm under constant temperature. This discrepancy indicates that rutting deformation occurring at earlier temperature levels enhances the density and rutting resistance of the DLAP, consequently influencing its future rutting behavior [32]. Figure 4b shows the appearance of the rutting specimen before and after loading under upward stepwise temperature. As can be seen from Figure 4b, the surface of asphalt mixture on both sides of the wheel track is flat, and there is no obvious bulge. This shows that the load effect on both sides of the wheel track is small when the temperature is low.

Table 5 shows the regression results for rutting deformation under upward stepwise temperature, where the power law was used to characterize the relationship between rutting deformation and load cycles. The values and standard errors of the regression parameters are presented in Table 5 to characterize their confidence intervals [35]. Furthermore, regression analysis of rutting development curves shows that rutting deformation at each temperature interval follows a power-law increase with load cycles (R^2^ > 0.97, Table 5). This indicates that rutting development under an increasing temperature state retains a certain regularity. Connecting rutting deformations at all temperature intervals by a specific method will contribute to further understanding rutting development regularity under variable temperature.

#### 3.1.3. Decreasing Temperature State

**Figure 5:**
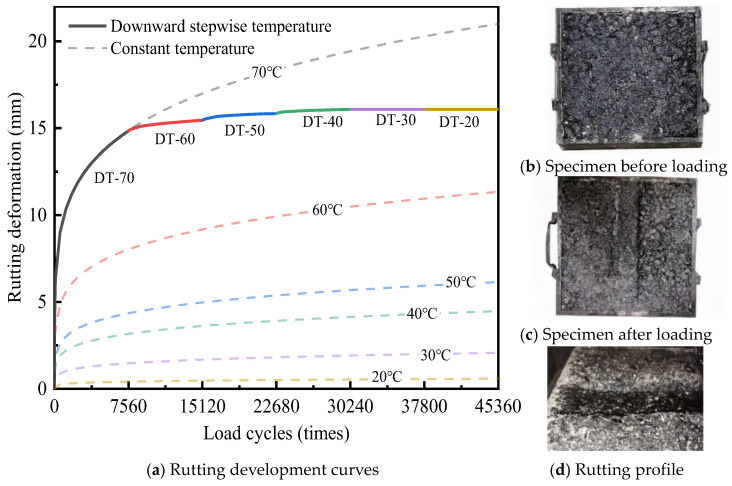
Rutting test results under downward stepwise temperature.

Figure 5 shows the rutting test results under downward stepwise temperature, where DT-20 indicates a decrease in temperature up to 20 °C.

The rutting development under downward stepwise temperature is relatively simple, as depicted in Figure 5a. Rutting deformation predominantly occurs during the high-temperature period, with 92.3% of the final rutting deformation occurring at 70 °C, followed by 3.9% at 60 °C, and 2.4% at 50 °C. The rutting deformation of DLAP reached a notable value of 15.8 mm at 50 °C. At this point, the DLAP had sufficient density, and load cycles at temperatures below 50 °C had a negligible impact on further rutting deformation. Specifically, rutting deformation at DT-40 increased by 0.23 mm, whereas negligible changes were observed at DT-30 and DT-20. These slight rutting deformations mainly originated from the creep characteristics of the asphalt mixtures. Figure 5b shows the appearance of the rutting specimen before and after loading under downward stepwise temperature. As can be seen from Figure 5b, the surface of the asphalt mixture on both sides of the wheel track shows obvious bumps. This shows that the loading effect on both sides of the wheel track is significant at high temperature.

Except for DT-30 and DT-20, rutting deformation also exhibited a power-law increase (R^2^ > 0.96) in different temperature intervals (Table 6). This result can be attributed to the negligible rutting development at DT-30 and DT-20, where rutting deformation showed little variation with load cycles, resulting in the absence of a functional relation.

### 3.2. Rutting Development Regularity Under Variable Temperature

#### 3.2.1. Rutting Test Result Under Variable Temperatures

**Figure 6:**
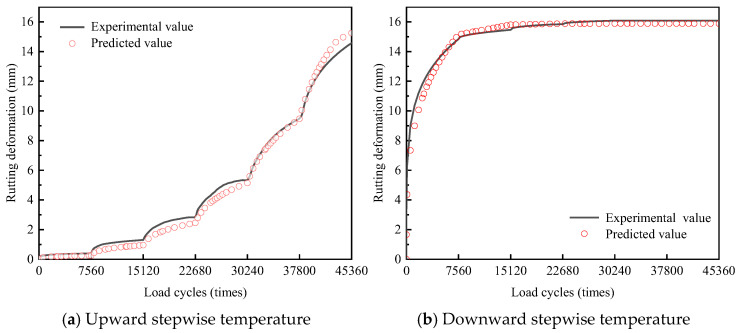
Predicted and experimental values from the rutting test.

The comparison between predicted and experimental values from rutting tests under variable temperature is shown in Figure 6.

As shown in Figure 6, the discrepancies between the predicted and experimental results are minimal. Under a downward stepwise temperature, the mean absolute error (MAE) and root-mean-square error (RMSE) between the predicted and experimental values are 0.29 mm and 0.43 mm, respectively, with a predicted error in final rutting deformation of 0.1%. Under an upward stepwise temperature, the discrepancies between the predicted and experimental values are slightly larger, with an MAE of 0.37 mm, an RMSE of 0.43 mm, and a predicted error in final rutting deformation of 0.8%. The primary reason is that lower temperatures enhance the anti-deformation ability of the asphalt mixture, thereby stabilizing deformation behavior and reducing prediction errors. Overall, there is a good agreement between the predicted and experimental values, demonstrating the feasibility of the time-hardening model for predicting rutting deformations under variable temperature.

#### 3.2.2. Verification of the Rutting Development Regularity

**Figure 7:**
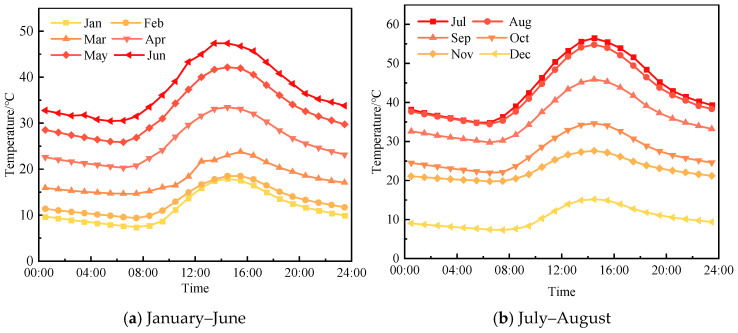
Annual variation in pavement temperatures under actual service conditions.

To validate the reliability of the time-hardening model in predicting rutting deformation, data on pavement temperature variation and traffic conditions were collected from the field test section. The annual standard axle-load repetition for the test section was approximately 219,000. The pavement temperature variation for the test section is shown in Figure 7.

**Figure 8:**
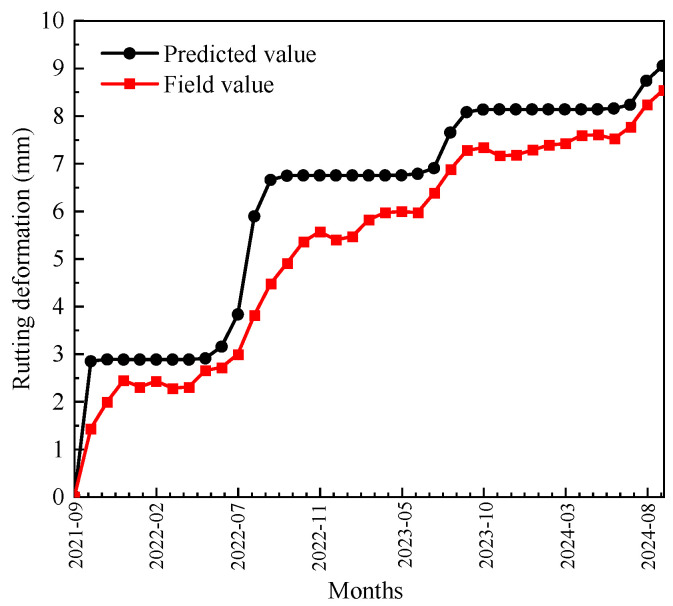
Comparison of predicted and field rutting deformation for the test section.

Using the time-hardening model, the rutting deformation after three years of operation can be predicted. A comparison of the predicted results with the field monitoring results is shown in Figure 8.

As shown in Figure 8, the trends of predicted and field rutting deformation are consistent, with rutting deformation gradually increasing over the months and exhibiting an S-shaped growth pattern. The growth rate of rutting deformation is uneven, displaying marked seasonal variation. In the high-temperature season (June to August), the rutting deformation of DLAP develops rapidly, with a larger error between predicted and field values during this period. This could be attributed to the significant temperature gradients within the DLAP during the summer, which were not accounted for in the rutting test. The neglect of temperature gradient variations limited the accuracy of the time-hardening model’s predictions. With the increase in months, the discrepancy between the predicted and measured values gradually decreases, stabilizing at 0.47 to 0.52 mm. During the initial stages, the DLAP was not densified under the load, making rutting growth sensitive to variations in pavement temperature and load. Consequently, the model struggles to accurately capture this complex variation, leading to larger errors. However, as the pavement gradually densifies over time, the rutting development rate stabilizes, allowing the model to predict rutting deformation with increasing accuracy.

### 3.3. Rutting Performance Evaluation of the DLAP

#### 3.3.1. Rutting Performance Under Actual Service Conditions

**Figure 9:**
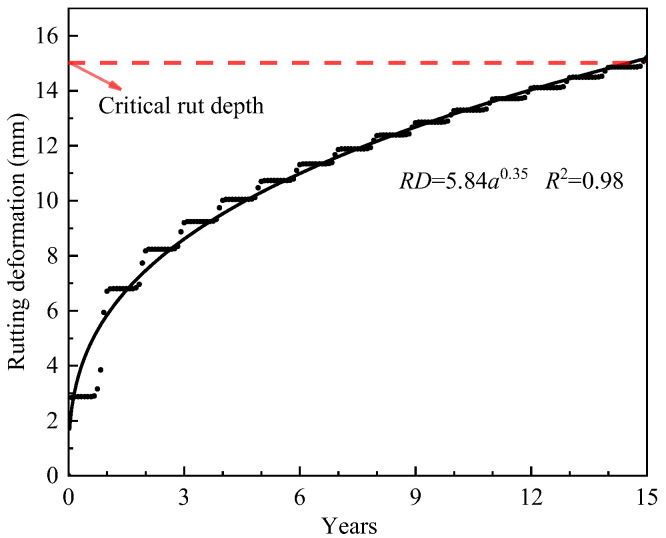
Rutting development curve under actual service conditions.

Based on the annual variation in pavement temperature (Figure 8), the rutting development of DLAP under actual service conditions was evaluated, with the results shown in Figure 9.

As shown in Figure 9, the rutting deformation curve exhibits a periodic S-shaped growth in an annual cycle, with the most significant development observed in June–August. During these months, the temperature of the pavement exceeds 30 °C, sometimes reaching 50 °C or more, which facilitates rapid rutting development. Nevertheless, this trend can still be fitted with the power function accurately. Rutting deformation primarily occurs in the first few years of traffic operation. Subsequently, the growth rate of rutting deformation decreases, with an annual increment of approximately 0.53 mm. At the end of the design life (15 years), the rutting deformation reaches the critical rutting depth of 15 mm. This suggests that the pavement structure utilizing DLAP is reasonable and can maintain stable road performance during the service period. However, DLAP may produce significant rutting during the early stages of traffic operation, which highlights the necessity for regular inspections and timely maintenance during this period [32]. Early preventive maintenance can effectively mitigate rutting development, enhance road performance, and reduce traffic accidents.

#### 3.3.2. Rutting Performance Under Extreme Heat Conditions

**Figure 10:**
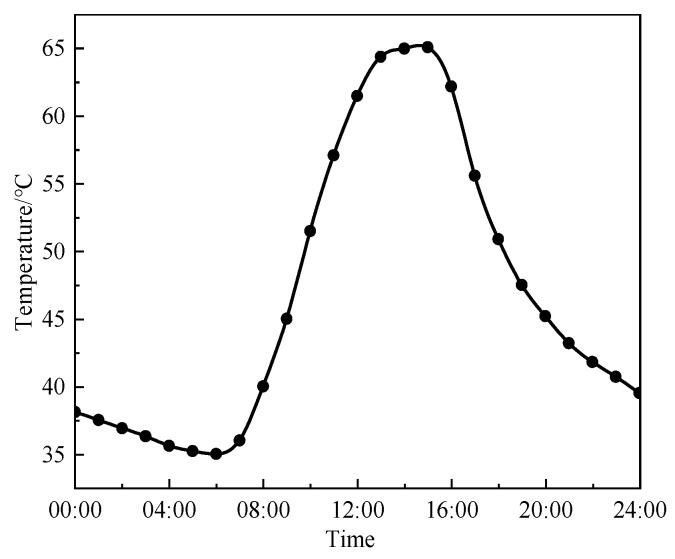
Daily variations in pavement temperatures under extreme heat.

**Figure 11:**
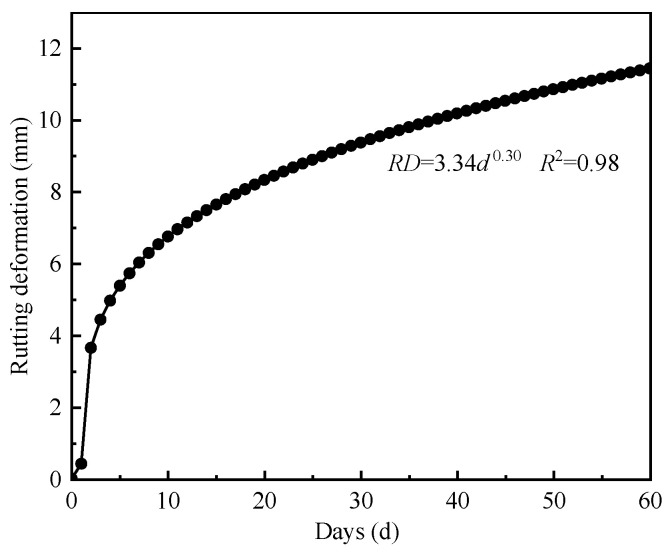
Rutting development curve under extreme heat conditions.

The field measured data indicate that the maximum pavement temperature in the test section can reach 65.1 °C, with daily temperature fluctuations as high as 30.1 °C (Figure 10). The rutting development of DLAP under extreme heat was analyzed using the time-hardening model, with the results presented in Figure 11.

As shown in Figure 11, the rutting deformation of DLAP increases following a power-law function as the duration of extreme heat increases. DLAP may develop severe rutting during initial exposure to extreme heat, with rutting deformation rapidly increasing to 3.65 mm. Subsequently, the growth rate of rutting deformation development significantly decreases. When the rutting deformation reaches 9 mm, the daily rutting deformation falls below 0.1 mm. This indicates that the DLAP gradually adapts to the extreme heat environment, forming a more stable internal structure, thereby significantly enhancing its ability to resist deformation. Typically, the duration of extreme heat is limited in summer. After 60 days of extreme heat, the rutting deformation of the DLAP still did not exceed the critical rut depth. This demonstrates that the pavement structure exhibits satisfactory reliability and can effectively withstand both the traffic load and high-temperature conditions in the test section.

## 4. Conclusions

In this study, a stepwise temperature-controlled rutting test method was proposed to investigate the effect of variable temperature on rutting development. A time-hardening model was developed and validated for predicting rutting deformation under variable temperature. Furthermore, the rutting performance of DLAP was evaluated based on actual pavement temperatures and traffic conditions. The following conclusions were drawn:

(1) The rutting deformation of DLAP exhibits a stepwise variation under variable temperature conditions, and the rutting development of DLAP demonstrates significant temperature dependence. When the temperature exceeds 50 °C, rutting deformation becomes uncontrollable and increases continuously at a high growth rate. It can be seen that the sensitive temperature for the high-temperature performance of DLAP is 50 °C. Therefore, the high-temperature performance of DLAP at 50 °C should be emphasized in the design of pavement materials.
(2) A time-hardening model was developed to predict the rutting deformation of DLAP under variable temperature based on stepwise temperature-controlled rutting test results. The prediction accuracy of the model was validated through field experiments. Stepwise temperature-controlled rutting tests on multilayer rutting specimens could offer an effective approach for estimating asphalt-pavement rutting deformation under variable temperature.
(3) The annual temperature variation of DLAP was collected on-site to evaluate its rutting performance. Under actual service conditions, the rutting development of DLAP can still be effectively described by the power-law function. The DLAP demonstrates satisfactory rutting resistance, which showing strong adaptability to the traffic loads and temperature conditions of the engineering practice.
(4) This study investigated the rutting performance of DLAP under variable temperature, achieving some preliminary results. However, the boundary conditions, loading conditions, and temperature gradients used in the rutting test differ from actual asphalt pavements. This discrepancy may limit the generalizability of the results. In future research, we will focus on the effect of temperature gradients and sensitive temperature ranges on the rutting deformation of rutting specimens in laboratory tests with high temperature, which will in turn modify the existing model and enhance its applicability.

## Figures and Tables

**Table 1:** Technical properties of the asphalts.

Asphalt	Penetration Index (0.1 mm)	Ductility (cm)	Soft Point (°C)	Relative Density	Dissolvability Intrichloro Ethylene (%)
70# petroleum asphalt	69	37	48.6	1.045	99.5
SBS-modified asphalt	65	70	85.0	1.035	99.6

**Table 2:** Technical properties of the aggregates.

Aggregate	Mineral Type	Technical Properties
Coarse aggregate	Amphibolite	Apparent density: 2.835 g/cm^3^; Crush value: 14.4%; Los Angeles attrition loss: 18.8%; Flakiness index: 6.3%
Fine aggregate	Limestone	Apparent density: 2.742 g/cm^3^; Methylene blue value: 3.1 g/kg; Sand equivalent: 82.3%; Angularity: 38.7 s
Mineral powder	Limestone	Apparent density: 2.717 g/cm^3^; Hydrophilic coefficient: 0.6; Plasticity index: 3.6%; Water content: 0.4%

**Table 3:** Mineral aggregate gradations of the asphalt mixtures.

Mixture Types	Mass Percentage Passing (%) for the Sieve Size (mm)											
	26.5	19	16	13.2	9.5	4.75	2.36	1.18	0.6	0.3	0.15	0.075
AC-16	-	100	94.3	88.2	70.3	45.0	33.2	22.7	16.1	9.9	7.3	5.8
AC-20	100	92.2	83.4	73.3	60.9	41.1	31.4	21.7	15.1	9.1	6.6	5.0

**Table 4:** Marshall test results for different asphalt concrete.

Mixture Types	VV (%)	VFA (%)	VMA (%)	MS (kN)	FL (mm)	OAC (%)
AC-16	3.4	73.2	14.2	13.3	3.3	4.5
AC-20	4.1	71.4	14.6	14.4	2.7	4.2

**Table 5:** Regression results for rutting deformation under upward stepwise temperature.

Regression Equation	Temperature Interval	Regression Parameter of *A*		Regression Parameter of *B*		*R* ^2^
		Value	Standard Error	Value	Standard Error	
*RD* = *AN^B^*	UT-20	0.070	0.016	0.193	0.001	0.99
	UT-30	0.226	0.008	0.196	0.004	0.99
	UT-40	0.516	0.016	0.194	0.004	0.99
	UT-50	1.121	0.056	0.177	0.006	0.97
	UT-60	1.744	0.069	0.188	0.005	0.99
	UT-70	4.280	0.132	0.136	0.004	0.98

**Table 6:** Regression results for rutting deformation under downward stepwise temperature.

Regression Equation	Temperature Interval	Regression Parameter of *A*		Regression Parameter of *B*		*R* ^2^
		Value	Standard Error	Value	Standard Error	
*RD* = *AN^B^*	DT-70	2.648	0.001	0.193	6.34 × 10^−6^	0.99
	DT-60	14.041	0.051	0.010	4.45 × 10^−4^	0.96
	DT-50	14.946	0.018	0.006	1.53 × 10^−4^	0.99
	DT-40	15.548	0.016	0.004	1.34 × 10^−4^	0.97
	DT-30	/	/	/	/	/
	DT-20	/	/	/	/	/

**Figure A1:**
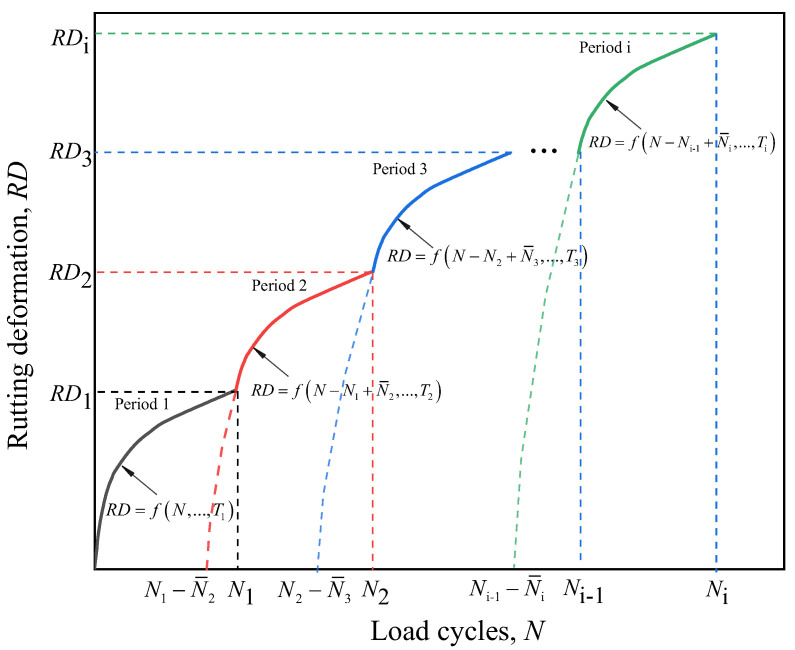
Demonstration of the time-hardening approach.

## Data Availability

The original contributions presented in this study are included in the article. Further inquiries can be directed to the corresponding author.

## Appendix A

### Appendix A.1. Formulation of the Rutting Development Regularity

The rutting test results indicate that rutting deformation accumulates with increasing load cycles, exhibiting a dependence on loading histories. Within each constant temperature interval, the rutting deformation exponentially increases with the number of load cycles. These rutting development patterns are similar to the characteristics of the time-hardening model for granular materials [36].

Based on the time-hardening model, the rutting development process under variable temperature is segmented into several periods [37]. For each period *i* (where *i* = 1, 2, 3, …), the average temperature *T_i_* represents the variational temperature within that interval. The relationship between rutting deformation, load cycles, and temperature under constant temperature can is described by Equation (A1):

(A1) $$RD=f\left(N,\ldots ,T\right)$$

Following the principle of deformation equivalence, the load cycles across adjacent temperature intervals are transformed as Equation (A2):

(A2) $${\bar{N}}_{i}=f^{-1}\left(RD_{i-1},\ldots ,T_{i}\right)$$

where ${\bar{N}}_{i}$ denotes the equivalent of load cycles under period *i* (*i* ≥ 2), and *RD_i_*_−1_ denotes the rutting deformation from the previous period *i* − 1.

The rutting development of asphalt pavement within period *i*, as it progresses through load cycles *N* (*N_i_*_−1_ < *N* ≤ *N_i_*), can be expressed as Equation (A3)

(A3) $$RD_{i}=f\left(N-N_{i-1}+{\bar{N}}_{i},\ldots ,T_{i}\right)$$

The calculation process for rutting deformation under variable temperature is illustrated in Figure A1. The rutting test results reveal the relationship between rutting deformation, load cycles, and temperature as Equation (A4):

(A4) $$RD=1.15\times 10^{-6}T^{3.27}N^{0.28}$$

The equivalent of load cycles under period *i* (*i* ≥ 2) can be represented as Equation (A5):

(A5) $${\bar{N}}_{i}={\left(\frac{RD_{i-1}}{1.15\times 10^{-6}T^{3.27}}\right)}^{3.57}$$

The development of rutting deformation within period *i* (*i* ≥ 2) can be represented as Equation (A6):

(A6) $$RD_{i}=1.15\times 10^{-6}T^{3.27}{\left(N+{\bar{N}}_{i}\right)}^{0.28}$$
